# Isolation and purification of potential weed inhibitors from *Mimosa pigra* L.

**DOI:** 10.1016/j.heliyon.2023.e18205

**Published:** 2023-07-17

**Authors:** Do Tan Khang, Tran Ngoc Quy, Nguyen Phuc Dam, Nguyen Trong Tuan, Tran Thanh Men, Nguyen Van Ay, Nguyen Phuong Thuy

**Affiliations:** a Institute of Food and Biotechnology, Can Tho University, 94000, Can Tho City, Viet Nam; b College of Education, Can Tho University, 94000, Can Tho City, Viet Nam; c College of Natural Sciences, Can Tho University, 94000, Can Tho City, Viet Nam; d College of Agriculture, Can Tho University, 94000, Can Tho City, Viet Nam; e School of Agriculture and Aquaculture, Tra Vinh University, Tra Vinh province, 87000, Viet Nam

**Keywords:** *Mimosa pigra*, Allelopathic, Inhibitor, *Echinochloa crus-galli*, Quercetin, Bio-herbicide

## Abstract

The diversity in structure and herbicidal properties detected in natural phytotoxic compounds could bring about advantages for development bio-herbicides. The present study was carried out search for potential weed inhibitors from the parts of *Mimosa pigra* L. The ethyl acetate (EtOAc) extract of leaf of *M. pigra* showed inhibitory activity during the time that *Echinochloa crus-galli* (barnyardgrass) germinates and grows, which is greater than that of other extracts. From this active extract, potent growth inhibitors were isolated and identified by column chromatography (CC), gas chromatography-mass spectrometry (GC-MS) and nuclear magnetic resonance (^1^H and ^13^C NMR). The six compounds were purified in this study namely: lupeol (C1, 13.2 mg), stigmastane-3,6-dione (C2, 14.7 mg), quercetin (C3, 20.2 mg), chrysoeriol (C4, 28 mg), methyl gallate (C5, 21.5 mg) and daucosterol (C6, 16.0 mg). The C2 (quercetin) compound completely inhibited the emergency, shoot height and root length of *E. crus-galli* at 1 mg/mL concentration (IC_50_ shoot height = 0.56 mg/mL). This was also the first study to report the isolation and allelopathic activity of lupeol, chrysoeriol and daucosterol from *M. pigra* leaf. Findings of this study highlighted that quercetin from *M. pigra* may become bio-herbicide to control barnyard grass and other grass weeds for the development of safe agriculture.

## 1 Introduction

Weeds are one of the most dangerous toxic groups in agricultural production along with animal pests, pathogens, and natural disasters. They cause serious damage up to 34% of the crops [1]. In paddy fields, weeds reduce the rice growth by competition for water, nutrients, light and space [2,3] Among them, barnyard grass (*Echinochloa crus-galli*) is the common problematic weed infesting rice fields in Vietnam [4,5]. It inhibits rice growth and reduces approximate 25% grain yield under high infestation condition [5]. Specifically, phenotype and morphology of barnyard grass are most similar with rice, so it is difficult to control or to kill [6].

Several years ago, there is a significant number of synthetic herbicides which have been used in agricultural production system [7]. Even though commercial herbicides recorded the high effectiveness in controlling weeds, these herbicides have resulted in an alteration related to the phytosociological composition of weeds and an increase in selecting herbicide-resistant weeds. On the other hand, the herbicides also cause negative influences on the environment as well as human health, which could not be underestimated [8,9]. Therefore, finding solutions or biological herbicides is necessary for the development of safe agriculture.

Allelopathy is known as a biological phenomenon in which an organism can produce phytochemicals that have adverse impact on the growth and development of other surrounding plant species [10]. These chemical components are considered as allelochemicals and able to be primarily generated as secondary metabolites derived from plants and microorganisms [11]. They can be synthesized in any plant parts such as roots, shoots, bark, leaves, flowers, seeds, etc. and released into the environment through root exudation, volatilization, decomposition and leaching processes [12]. As natural toxins, allelochemicals have been employed as a substitute for commercial herbicides and chemical control applications for the eradication of weed plants in agroecosystems [13,14].

*Mimosa pigra* L is a large shrub native to tropical America and naturalized in tropical [15]. It has been described as an invasive weed and often threatens biodiversity [16]. *M. pigra* is characterized by a strong ability to grow and its seeds can exist a long time in the environment. Currently, it is one of 100 species of dangerous invasive plants [17] and is becoming a serious threat to ecosystems, invading many regions of many countries as well as Vietnam. Many biological compounds belonging to the groups of flavonoids, polyphenols, glycosides, and lignans have been found in the leaves of *M. pigra*. Recently, Intira Koodkaew [15] reported that methanol extract of *M. pigra* has been capable of inhibiting the growth of two tested weeds; however, the components which are active in this activity seem to be doubtful.

This study therefore carried out the bioassays involving in germination and growth of *M. pigra* which inhibits the growth of *Echinochloa crus-galli* regarding to various parts, extracting solvents and fractional stages compartmentalized by using column chromatography (CC). In addition, gas chromatography-mass spectrometry (GC-MS) and nuclear magnetic resonance (^1^H and ^13^C NMR) are common methods whereby the chemical structures of isolated compounds were determined. The levels of inhibition detected in purified constituents from *M. pigra* were also examined and characterized.

## 2 Materials and methods

### 2.1 Chemicals

Methanol, hexane, ethyl acetate, dichloromethane, ethanol, iso-butanol, diethyl ether was obtained from the CHEMSOL VINA company, Vietnam.

### 2.2 *M. pigra* materials

The different parts of *M. pigra* (roots, bark, leaves, flowers, fruits, and seeds) were collected in the wasteland around Can Tho city. They were dried by freeze-drying machine at 40 °C. The dried and sterilized samples were pulverized to a fine powder using a grinding machine.

The dry powder of each component is weighed in 10 g, put into 100 mL of methanol and soaked for 3 weeks to extract phytochemical constituents. The sample is then filtered through cloth and filter paper. The result is methanol extract corresponding to each sample. The methanol extract is placed in a glass vial with a lid and stored in a refrigerator at an average temperature of 3–5 °C. After that, each sample was put into the rotary evaporator to get the extract.

### 2.3 Preparation extracts of *M. pigra*

The powder (leaf, 1.0 kg) was soaked in 10 L methanol (MeOH) for 3 weeks to collect bioactive components at room temperature. After filtration, the filtrate from powder-methanol dispersion was concentrated under vacuum at 50 °C using a rotary evaporator to produce 80 g of crude extract (3 days) [18]. Subsequently, the crude extract with greatest inhibition was serially fractionated with hexane and ethyl acetate (EtOAc) after being diluted in distilled water (300 mL). After screening the allelopathic activities, the bioactive compounds were isolated with the EtOAc extract by using column chromatography (Fig. 2).

### 2.4 Compound isolation from ethyl acetate extract

The ethyl acetate extract was fragmented in a normal phase of column (600 mm height × 40 mm diameter) filled with silica gel (200–400 mesh particle size). This extract was fractionated by increasing the polarity with following eluents: Hex 100%, Hex:EA (80:20), Hex:EA (50:50), EA 100%, EA:MeOH (50:50) and MeOH 100%.

All fractions were performed by thin-layer chromatography (TLC) and further separated according to the respective solvent by column chromatography filled with silica gel (70–230 mesh particle size) to get compounds. The compounds were crystallized and purified with the respective solvents: dichloromethane, ethanol, iso-butanol, diethyl ether. Crystal compounds or pure compounds were confirmed by TLC of 90.00% and then they are sent to measure the spectrum.

The structures of compounds were identified by spectroscopic methods: ^1^H and ^13^C NMR at Vietnam Academy of Science and Technology (Bruker BioSpin, Switzerland).

### 2.5 Germination and growth bioassays

The herbicidal assays were evaluated using the protocol described by Xuan et al. [19]. The sample solutions (5 mL, crude extracts, fractions or compounds) were loaded in each Petri dish respectively. The control treatment does not use any extract, using methanol solvent. The Petri dishes are placed in a fume cupboard for 20 h to allow the methanol to evaporate completely, leaving only the extract on the filters. After methanol evaporated, a total of 10 healthy seeds of barnyard grass (*Echinochloa crus-galli*) were placed and added 3 mL of distilled water. The photoperiod of growth chamber was 12/12 h day/night with temperature 25 °C. After 5 days, germination rate, shoot height, root length, fresh weight and dry weight were evaluated. The percentages of germination, shoot, root, fresh weight, and dry weight over the control were indicated as the inhibitory percentage (%).

### 2.6 Statistical analysis

The study used a randomized block design experimental method with three times replications. The collected data was presented as the average ± standard deviation from triplicate determinations. The statistical analysis was conducted in one-way ANOVA using Minitab® 16.0 (©2012 Minitab Inc.; Philadelphia, PA, USA). The results were displayed as means ± standard deviation (SD) values. The significant difference of treatments were examined by using Turkey’s test with the confidence level of 95% (p < 0.05).

## 3 Results

### 3.1 Inhibitory effect of extracts from different parts of *M. pigra*

The various inhibitory levels of extracts derived from different parts of *M. pigra* (root, shoot, leaf, fruit, flower, seed) was shown in Table 1. Most of these extracts inhibited germination of barnyard grass at all concentrations. Germination inhibition rates ranged from 0.03 to 34.51%. However, most of the treatments were not statistically significant difference at the 5% level. The ability to inhibit root growth was highest in the seed extract (5 mg/mL) at 76.19%; followed by leaf extract (5 mg/mL) at 71.17%. Shoot growth was inhibited by leaf and flower extracts at a dosage of 5 mg/mL with rates of 61.60% and 61.67%, respectively. Additionally, fresh weight was also inhibited by these two extracts with 56.69% and 57.50%.

**Table 1:** Inhibitory efficacy of extracts derived from different parts of *M. pigra* on germination and growth of *Echinochloa crus-galli*.

Treat.	Conc. (mg/ml)	Germination (%)	Inhibition percentage (%)			
			Root	Shoot	Fresh weight	Dry weight
Root	1.0	13.82 ± 5.97^a^	44.37 ± 0.24^f^	45.25 ± 0.54^ef^	46.09 ± 1.91^bcd^	18.75 ± 1.02^bc^
Root	2.5	10.38 ± 5.97^a^	62.14 ± 1.62^d^	43.69 ± 0.72^ef^	48.66 ± 13.80^abc^	16.68 ± 0.64^c^
Root	5.0	6.93 ± 10.34^a^	63.63 ± 0.72^d^	43.14 ± 0.47^fg^	45.13 ± 0.20^bcde^	14.10 ± 0.39^d^
Shoot	1.0	10.38 ± 15.79^a^	44.71 ± 1.66^f^	32.22 ± 1.22^i^	40.35 ± 0.09^cdef^	7.66 ± 0.70^fg^
Shoot	2.5	3.41 ± 0.00^a^	50.95 ± 6.66^e^	41.11 ± 1.02^gh^	38.45 ± 0.04^def^	0.55 ± 0.44^hi^
Shoot	5.0	10.38 ± 15.80^a^	66.14 ± 0.51^bcd^	46.40 ± 0.42^de^	35.44 ± 0.08^ef^	1.97 ± 0.74^h^
Leaf	1.0	3.48 ± 5.97^a^	44.77 ± 0.82^f^	49.12 ± 1.06^cd^	43.39 ± 0.11^bcdef^	−1.20 ± 0.83^i^
Leaf	2.5	0.03 ± 5.97^a^	52.71 ± 0.77^e^	52.17 ± 1.43^b^	51.93 ± 0.28^ab^	6.82 ± 0.15^g^
Leaf	5.0	3.48 ± 5.97^a^	71.17 ± 0.77^ab^	61.60 ± 1.53^a^	56.69 ± 0.03^a^	13.81 ± 0.92^d^
Fruit	1.0	6.93 ± 10.30^a^	14.58 ± 0.47^i^	44.84 ± 0.71^ef^	35.62 ± 0.15^ef^	−7.40 ± 0.24^j^
Fruit	2.5	0.03 ± 5.97^a^	27.00 ± 1.43^h^	43.35 ± 0.24f^g^	34.25 ± 0.10^f^	7.53 ± 0.56^g^
Fruit	5.0	0.03 ± 5.97^a^	42.39 ± 0.93^f^	50.54 ± 0.93^bc^	41.62 ± 0.11^cdef^	7.63 ± 1.02^fg^
Flower	1.0	6.93 ± 10.34^a^	15.12 ± 0.60^1i^	43.28 ± 0.31f^g^	51.93 ± 0.06^ab^	19.85 ± 1.21^b^
Flower	2.5	3.48 ± 5.97^a^	34.05 ± 0.00^g^	48.64 ± 0.77^cd^	52.10 ± 0.07^ab^	20.24 ± 0.83^b^
Flower	5.0	13.82 ± 5.97^a^	65.26 ± 0.77^cd^	61.67 ± 0.92^a^	57.50 ± 0.10^a^	23.12 ± 0.24^a^
Seed	1.0	6.93 ± 0.34^a^	43.28 ± 0.24^f^	39.55 ± 1.06^h^	46.12 ± 0.09^bcd^	9.73 ± 0.06^ef^
Seed	2.5	6.93 ± 0.00^a^	70.22 ± 0.47^bc^	52.98 ± 0.20^b^	33.63 ± 0.16^f^	9.96 ± 1.09^e^
Seed	5.0	17.27 ± 0.00^a^	76.19 ± 0.81^a^	44.30 ± 1.23^ef^	46.20 ± 0.16^bcd^	24.09 ± 0.53^a^

Values represent means ± SD (standard deviation). Values with different superscript letters in a column were significant different according to Turkey’s test (p < 0.05). “-” represents the stimulant effect on experimental weed.

**Fig. 1:**
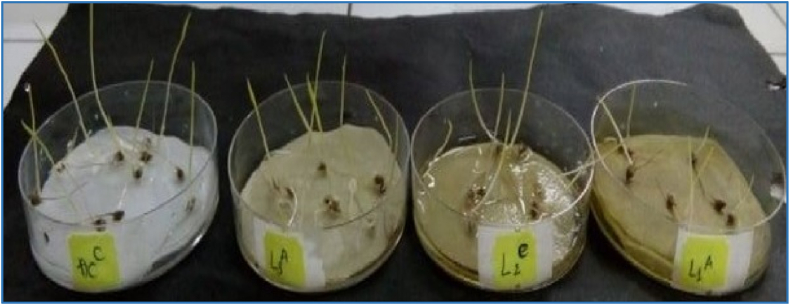
Effect of leaf extract of *M. pigra* on germination and growth of barnyardgrass (ĐC) Control (L1, L2, L3) Leaf.

In general, the leaf extract of *M. pigra* had highest inhibitory capacity on the shoot height and root length of barnyardgrass, so the leaf extract was selected to continuously conduct experiments for further fractionation (Fig. 1).

**Fig. 2:**
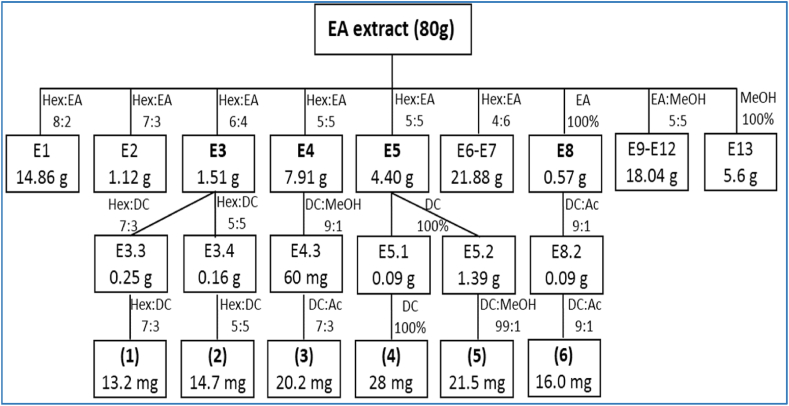
Procedure of isolation of bioactive compounds from EtOAc extract from leaf of *M. pigra* (HE) Hexane (EA) Ethyl acetate (MeOH) Methanol.

### 3.2 Inhibitory effect of crude extracts from leaf of *M. pigra*

The inhibitory levels of leaf extract with different polar solvents (hexane, ethyl acetate and water) was displayed in Table 2. It was found that the ethyl acetate (EtOAc) extract exhibited the highest inhibition of germination and growth of barnyard grass and inhibitory activities were in direct proportion to the applied doses. In particular, the EtOAc fraction of 0.5 mg/mL concentration completely inhibited the root length of *E. crus-galli*, followed by germination (95%), shoot length (71.35%) and fresh weight (60.62%) (Table 2).

**Table 2:** Effect of crude extracts from leaf of *M. pigra* on germination and growth of *Echinochloa crus-galli*.

Treatments	Conc. (mg/ml)	Germination (%)	Inhibition percentage (%)			
			Root	Shoot	Fresh weight	Dry weight
Hexane	0.10	55.02 ± 14.99^bc^	−8.17 ± 1.01^g^	10.67 ± 0.49^f^	−23.79 ± 6.40^f^	9.61 ± 0.23^g^
Hexane	0.25	70.01 ± 25.97^abc^	14.96 ± 1.91^f^	17.46 ± 2.14^e^	−40.87 ± 0.67^g^	13.14 ± 2.55^f^
Hexane	0.50	75.01 ± 8.66^abc^	48.23 ± 0.38^d^	44.52 ± 0.83^d^	19.62 ± 0.08^d^	37.57 ± 0.23^c^
EtOAc	0.10	80.01 ± 22.90^abc^	23.99 ± 3.03^e^	14.54 ± 1.55^ef^	9.89 ± 0.36^e^	24.11 ± 2.16^d^
EtOAc	0.25	85.01 ± 14.99^ab^	89.42 ± 1.32^b^	47.05 ± 0.83^d^	55.26 ± 0.76^ab^	16.54 ± 0.39^e^
EtOAc	0.50	95.00 ± 4.64^a^	100.00 ± 0.00^a^	71.35 ± 0.71^a^	60.62 ± 2.80^a^	22.15 ± 0.60^d^
Water	0.10	50.02 ± 22.90^c^	10.55 ± 3.64^f^	58.02 ± 0.36^c^	27.41 ± 0.05^d^	50.10 ± 0.99b
Water	0.25	55.02 ± 14.99^bc^	74.22 ± 1.14^c^	63.46 ± 4.80^b^	53.21 ± 0.21^c^	67.09 ± 0.39^a^
Water	0.50	90.00 ± 17.31^ab^	95.59 ± 0.76^a^	67.57 ± 0.85^ab^	53.57 ± 0.17^ab^	65.13 ± 0.39^a^

Values represent means ± SD (standard deviation). Values with different superscript letters in a column were significant different according to Turkey’s test (p < 0.05). “-” represents the stimulant effect on experimental weed.

Besides, the inhibition of weed growth of the water fraction was quite high at root length (95.59%) and this fraction is not statistically different from the EtOAc fraction at 5% significance level. It is assumed that the greater inhibition of the EtOAc extract which means it contains more potent allelochemicals than the other fractions. Thus, this fraction was chosen for column chromatography to isolate bioactive compounds.

### 3.3 Isolation and purification of plant growth inhibitors by column chromatography

The bioactive compounds containing in the EtOAc extract from the leaf of *M. pigra* were isolated by using column chromatography (Fig. 2). Different combinations of solvents were used, of which the mixture consisting of hexane (HE) and ethyl acetate (EA) recorded the highest efficacy in isolating and separating the components of the EtOAc extract. Ultimately, the combinations of H:E from 6:4 to 0:10 successfully compartmentalized six compounds identified as C1–C6 (Fig. 2, Table 3).

**Table 3:** Identification of bioactive compounds from EtOAc extract from leaf of *M. pigra* by GC-MS and ^1^H and^13^C NMR.

Fractions	Solvent (hexane:ethyl acetate)	Weight (mg)
C1	Crystal in HE-EA 6:4	13.2
C2	Crystal in HE-EA 6:4	14.7
C3	Crystal in HE-EA 5:5	20.2
C4	Crystal in HE-EA 5:5	28.0
C5	Crystal in HE-EA 5:5	21.5
C6	Crystal in HE-EA 0:10	16.0

### 3.4 NMR structural elucidation

Compound (**1**) was isolated from EA fraction as white needles. The positive ESI-MS spectrum of (**1**) showed an ion peak at *m/z* 409 [M + H–H_2_O]^+^, indicating molecular formula of this compound as C_30_H_50_O. The ^1^H NMR indicated seven single signals of methyl groups at δ_Hppm_0.76, 0.79, 0.83, 0.95, 0.97, 1.03 and 1.66. Besides, the presence of one oxygenated methine at δ_Hppm_3.19 (1H, *dd*, *J* = 11.5, 5.0 Hz, H-3), two olefin protons of CH_2_ = group at δ_Hppm_4.57 (1H, *brs*, H-29a) and 4.69 (1H, *brs*, H-29b). The ^13^C NMR and DEPT spectra showed characteristic signals of a triterpenoid skeleton with total 30 carbons, including twoolefin carbons at *δ*_Cppm_ 151.0 and 109.3, one oxygenated methine carbon at δ_Cppm_79.0, and seven methyl carbons at δ_Cppm_14.6, 15.4, 16.0, 16.1, 18.0, 19.3 and 28.0. These spectra data indicated (**1**) possessed the lupane skeleton with one hydroxyl group at position C-3. Thus, the structure of compound (**1**) was identified as lupeol based on these NMR analysis and comparison with reported publication [20].

Compound (**2**) was obtained as white needles. Its molecular formula was identified as C_29_H_48_O_2_ by an ion peak at *m/z* 429 [M+H]^+^ in (+) ESI-MS spectrum. The ^1^H NMR showed seven methyl groups including two single signals at *δ*_Hppm_ 0.69 and 0.96, three doublet signals at δ_Hppm_0.81, 0.83 and 0.94, and one triplet signal at δ_Hppm_0.85. The ^13^C NMR and DEPT spectra showed 29 characteristic carbons of a steroid skeleton with seven methyl carbons at *δ*_Cppm_ 12.0,12.2, 12.6, 18.7, 19.0 and 19.8. Besides, there were two carbonyl carbons at *δ*_Cppm_ 209.1 and 212.2 indicating that the presence of two ketone groups in its structure. These two ketone was located at position C-3 and C-6 confirmed by HMBC correlations between methylene protons of H-2, H-4 at *δ*_Hppm_ 2.33–2.41 (2H, *m*, H-2), 2.59 (1H, *m*, H-4a), 2.32 (1H, *m*, H-4b) and carbonyl carbon at *δ*_Cppm_ 211.2 (C-3), together with the cross peaks between methylene protons of H-7 at *δ*_Hppm_ 2.39 (1H, *m*, H-7a), 2.00 (1H, *t*, 13.0 Hz, H-7b) and carbonyl carbon at *δ*_Cppm_ 209.1 (C-6). Then, the result from comparison of its data with the research [21] revealed the structure of compound (**2**) which was similar to stigmastane-3,6-dione.

**Fig. 3:**
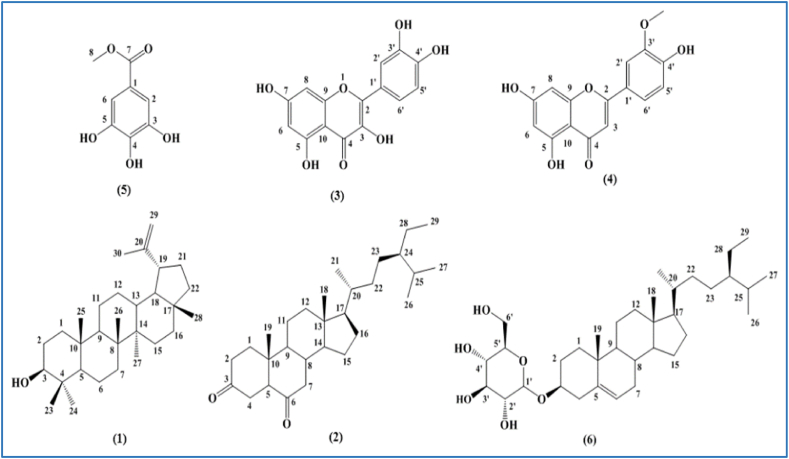
Chemical structures of bioactive constituents from *M. pigra*. (1) Lupeol (2) Stigmastane-3,6-dione (3) quercetin (4) Chrysoeriol (5) Methyl gallate (6) Daucosterol.

Compound (**3**) was isolated as yellow needles with the molecular formula C_15_H_10_O_7_, confirmed by the (−) ESI-MS spectrum with an ion peak at *m*/*z*: 301 [M − H]^-^. The ^1^H NMR showed five aromatic protons including two meta protons at *δ*_Hppm_ 6.18 (1H, *d*, *J* = 1.5 Hz, H-6) and 6.40 (1H, *d*, *J* = 2.0 Hz, H-8) of ring A, three aromatic protons of an ABX system at δ_Hppm_7.67 (1H, *d*, *J* = 2.5 Hz, H-2′), 6.89 (1H, *d*, *J* = 8.5 Hz, H-5′), 7.54 (1H, *dd*, *J* = 8.5, 2.0 Hz, H-6′) of ring B of a flavonoid skeleton. The ^13^C NMR and DEPTspectra also showed the typical flavonoid skeleton with 15 carbons from 93 to 180 ppm, including one carbonyl carbon at *δ*_Cppm_ 175.8, five methine aromatic carbons at *δ*_Cppm_ 98.1, 93.3, 115.0, 115.6 and 119.9 and nine quaternary carbons at *δ*_Cppm_ 147.7, 135.7, 160.7, 163.8, 156.1, 145.0, 146.8, 103.0 and 121.9. Based on the spectroscopic data and compared to the previous study [22], the structure of compound (**3**) was elucidated as quercetin (Fig. 3).

Compound (**4**) was collected as pale-yellow powder; its molecular formula was C_16_H_12_O_6_ which was deduced by the (−) ESI-MS spectrum with an ion peak at *m*/*z*: 299 [M − H]^-^. The ^13^C NMR and DEPT spectra of compound (**3**) also showed characteristic signals of a flavonoid skeleton with 15 carbons from 93 to 180 ppm. The ^1^H NMR spectrum showed the signals of five aromatic protons including two protons at *δ*_Hppm_ 6.20 (1H, *d*, *J* = 2.0 Hz, H-6), and 6.51 (1H, *d*, *J* = 2.0 Hz) of ring A of the flavonoid; and three protons at *δ*_Hppm_ 7.55 (1H, *m*, H-2′), 6.94 (1H, *d*, *J* = 9.0 Hz, H-5′), and 7.56 (1H, *m*, H-6′) of an ABX system of ring B. In addition, one olefin proton appeared at *δ*_Hppm_ 6.89 (1H, *s*, H-3). This analysis revealed the structure of compound (**3**) as a luteolin derivative. Besides, compound MD01 has one oxygenated methyl group at *δ*_Hppm_ 3.89 (3H, *s*, 3′-OCH_3_) and *δ*_Cppm_ 55.9 in its structure. The HMBC spectrum described the molecular correlation between methyl group at *δ*_Hppm_ 3.89 (3H, *s*) and carbon C-3′ of quercetin at δ_Cppm_148.0 indicating the position of methyl group at C-3′. Therefore, compound (**4**) was characterized as chrysoeriol [23].

Compound (**5**) was extracted as amorphous white powder. The ESI-MS spectrum of (**1**) in negative mode showed an ion peak at *m/z* 183 [M − H]^−^, delineating its molecular formula as C_8_H_8_O_5_. The ^13^C NMR spectrum of compound (**1**) showed signals of one carbonyl carbon at *δ*_C ppm_ 167.3 and one oxygenated methyl carbon at *δ*_C ppm_ 51.9 revealed the presence of methyl ester group. In addition, six aromatic carbons appeared from *δ*_C ppm_ 105–150, in which there were two pairs of equivalents carbons at *δ*_C ppm_ 109.8 and 146.1, two quaternary carbons at *δ*_C ppm_ 121.7 and 138.8 belonging to a 3,4,5-trisubsituted benzoic acid derivative. The ^1^H NMR demonstrated one signal of two aromatic protons at *δ*_H ppm_ 7.10 (2H, s) and one methyl group at *δ*_H ppm_ 3.78 (3H, s). From these evidence and in comparison, with previous publication, compound (**5**) was identified as methyl gallate [24].

Compound (**6**) was isolated from EA fraction as white powder and determined as daucosterol by comparison the NMR data of this compound and previous publication [23].

### 3.5 Inhibitory effect of puried compounds from ethyl acetate extraction of *M. pigra*

The phytotoxic effects of the isolated compounds, which were examined throughout the process when barnyard grass germinates and grows, was shown in Table 4. In general, all of them had inhibition capacity and the inhibitory levels were proportional to the applied concentrations. In particular, C3 totally inhibited the germination and elongation of roots and shoots of this weed which was detected at the concentration of 1 mg/mL (Table 4). Simultaneously, 1 mg/mL is the concentration that all compounds illustrated the high inhibitory capacity in root length (>87%). At the same applied concentration, the inhibitory magnitudes of C1, C3, and C4 also displayed much greater inhibition on the shoot length of *E. crus-galli* than other compounds*.* However, all of them present low inhibitory level in germination of barnyard grass except C3.

**Table 4:** Effect of puried compounds of *M. pigra* on germination and growth of *Echinochloa crus-galli*.

Compounds	Concentration (mM)	Inhibition percentage (%)		
		Shoot	Root	Germination
		16.67 ± 12.10^fgh^	39.47 ± 25.47^cd^	6.67 ± 5.77^fg^
	0.5	42.44 ± 11.09^de^	34.17 ± 6.57^d^	16.67 ± 5.77^defg^
	1.0	84.30 ± 9.71^abc^	89.54 ± 3.65^a^	33.33 ± 5.77^bcd^
C2	0.1	10.85 ± 8.88^fgh^	25.38 ± 22.37^de^	10.00 ± 10.00^efg^
	0.5	35.27 ± 5.08^efg^	42.40 ± 3.95^cd^	23.33 ± 5.77^cdef^
	1.0	71.32 ± 3.78^bc^	87.59 ± 6.49^ab^	36.67 ± 5.77^bc^
C3	0.1	36.05 ± 4.76^ef^	46.03 ± 7.35^bcd^	20.00 ± 10.00^cdef^
	0.5	64.92 ± 1.87^cd^	81.59 ± 10.85^abc^	36.67 ± 5.77^bc^
	1.0	100.00 ± 0.00^a^	100.00 ± 0.00^a^	100.00 ± 0.00^a^
C4	0.1	11.05 ± 6.04^fgh^	8.51 ± 4.81^de^	6.67 ± 5.77^fg^
	0.5	29.07 ± 16.06^efgh^	36.40 ± 12.73^d^	16.67 ± 5.77^defg^
	1.0	91.86 ± 1.54^ab^	92.61 ± 0.64^a^	23.33 ± 5.77^cdef^
C5	0.1	9.50 ± 10.42^h^	28.87 ± 18.24^de^	13.33 ± 5.77^efg^
	0.5	12.60 ± 10.57^fgh^	37.38 ± 36.43^d^	23.33 ± 5.77^cdef^
	1.0	76.36 ± 5.24^abc^	93.58 ± 1.58^a^	26.67 ± 5.77^cde^
C6	0.1	24.03 ± 5.50^fgh^	19.53 ± 8.27^de^	0.00 ± 0.00^g^
	0.5	19.38 ± 7.74^efgh^	−12.41 ± 5.80^e^	13.33 ± 5.77^efg^
	1.0	70.16 ± 7.94^bc^	88.70 ± 6.91^a^	33.33 ± 5.77^bcd^

Values represent means ± SD (standard deviation). Values with different superscript letters in a column were significant different according to Turkey’s test (p < 0.05).

**Fig. 4:**
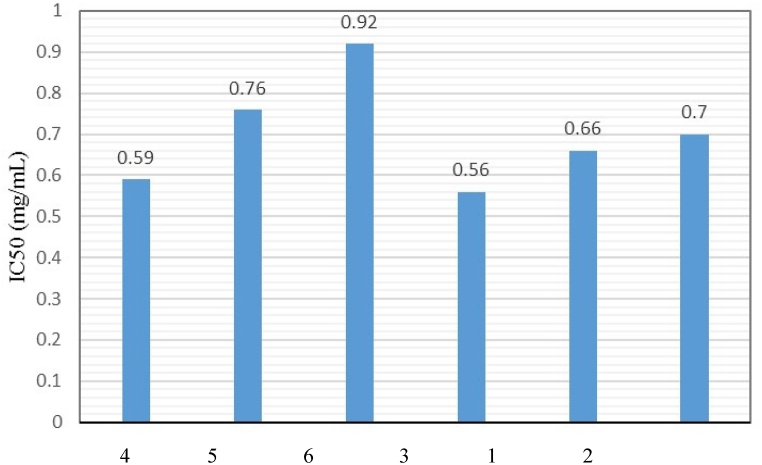
Effect of bioactive constituents from *M. pigra* on the shoot length of barnyardgrass (1) Lupeol (2) Stigmastane-3,6-dione (3) quercetin (4) Chrysoeriol (5) Methyl gallate (6) Daucosterol.

The inhibition on the processes related to germination and growth of barnyard grass was also proven by the IC_50_ value (the concentration inhibits 50% of the shoot height and a lower IC_50_ shows a higher inhibitory activity). Generally, the IC_50_ value of C3 (quercetin) is the lowest (0.56 mg/mL) as compared with other compounds. The finding in Table 4 and Fig. 4 suggest that quercetin may possess a novel mode of inhibitory action on *E. crus-galli*.

## 4 Discussion

The utilization of herbicide is a consideration in modern agriculture, which is effective way to restrict the growth of weeds that put a pressure threatening the productivity of crop [25,26]. However, these chemicals might bring some impacts on ecosystems as well as human health [27,28]. As a result, the weed management should pay a significant attention on developing some environmentally friendly methods in order to reduce the dependence on herbicide products which have already been in commercial [29]. The detected natural phytotoxic constituents could be a potential in assisting the of both directly used natural phytotoxins and synthetic herbicides [30,31].

This research showed that leaf extract of *M. pigra* had the more prominent inhibitory effects on the germination and growth of *Echinochloa crus-galli*. Similar to this result, Koodkaew and Rottasa [17] also reported that *M. pigra* leaf powder inhibited the germination and growth of *Ruellia tuberosa* L. Remarkably, Koodkaew and Wannathong age [32], revealed that *M. pigra* leaf extract inhibited the growth and reduced the chlorophyll accumulation of *Echinochloa crus-galli*. More specifically, the percentage involving in the inhibitory activity followed an upward trend according to the increase in concentration of the extract.

In this research, EtOAc extraction from leaf of *M. pigra* had the highest inhibitory efficacy on the germination, shoot height and root length of barnyardgrass, in comparison with hexane, and aqueous residues (Table 2). It is convinced that EtOAc extract of *M. pigra* leaf might contain potential allelochemicals. Li et al. [33] also indicated that suitable solvents can achieve great yields of potent allelochemicals*.*

The current study also mentioned the isolation, separation, and identification of phytotoxic compounds from *M. pigra* (Fig. 2; Table 3), suggesting a detailed description of herbicidal properties of *M. pigra* with five compounds including lupeol, stigmastane-3,6-dione, quercetin, chrysoeriol, and daucosterol (Table 4). Among them, quercetin is the major flavonoid compound presenting in more 20 plants in nature [34].

In previous research, Nasir et al. [35] isolated and identified compounds from flower extracts of *Robinia pseudo-acacia*. From the nuclear magnetic resonance (NMR) and mass spectroscopy analysis, the ethanolic extract had 3 active compounds including robinetin, myricetin and quercetin. All of them inhibited the shoot and root length of lettuce by 50% at a concentration of 100 ppm. Besides, Fernando et al. [35] tested the effect of leaf extract of *Leonurus sibiricus* L. on *Raphanus sativus*, *Lactuca sativa* and *Lepidium sativum*. The flavonoid compounds identified from this extract include quercetin-3-*O*-a-l-rhamnopyranosyl-(1 > 6)-b-d-galactopyranoside, rutin, hyperin, isoquercetrin, genkwanin, 3′-hydroxy genkwanin and quercetin. Concretely, 3′–OH–genkwanin and quercetin showed the stronger germination inhibition at concentrations of 10^−4^ M, meanwhile isoquercetrin, 3′–OH–genkwanin and rutin significantly inhibited root and shoot elongation.

The allelopathic activity of quercetin on grasses and weeds was further confirmed by many previous researchers. Rajyalakshmi et al. [36] isolated and purified constituents from *Nerium oleander* L. and investigated the influences on the growth of *Parthenium hysterophorus* L. From high-performance liquid chromatography (HPLC), quercetin was identified and significantly inhibited the germination, root and shoot length of *Parthenium hysterophorus* at the concentration of 6 mg/g.

Dhanya and Benny [37] isolated and investigated the herbicidal activity of the leaf extract of *Garcinia gummi – gutta*. The results showed that the extract at 75% concentration inhibits 50% of the germination rate of *Cicer arietnum, Pisum sativum, Cicer arietnum, Arachis hypogea and Vigna radiata*. After purification and determination of molecular structure by liquid chromatography-mass spectrometry (LC-MS) and NMR spectroscopy, it was confirmed that allelopathic compound was quercetin.

Kaab et al. [38] also revealed that quercetin is one of five compounds isolating from methanolic extract of *Cynara cardunculus*. It inhibited weed germination and seedling growth of *Trifolium incarnatum*, *Silybum marianum* and *Phalaris minor*. Furthermore, the crude methanolic extract *C. cardunculus* showed the same allelopathic activity as pelargonic acid (commercial bioherbicide compound). Kaab et al. [39] observed that crude extract of *C. cardunculus* induces oxidative stress and disorders the biochemical and physiological functions of the plant cells.

The report of Latif et al. [40] which was conducted under net-field condition, indicated that foliar tissues and rhizosphere soils of pasture legumes with high flavonoid content exhibit marked phytotoxicity on *Lepidium sativum* L. and *Lolium rigidum*. Concretely, the percentage inhibition ranged from 22 to 76% at a concentration of 5 mg/cm^3^, and from 27 to 96% by double (10 mg/cm^3^). IC_50_ analysis showed that with concentrations fluctuated from 0.6 to 4.21 mg/mL, the extract inhibited 50% of root growth and embryo axis. Based on chemometric analyses, the authors also indicated that quercetin, isoquercetin, kaempferol, and kaempferol-7-*O*-glucoside have strong weed inhibitory activity under field conditions.

In this research, phytochemical investigation of leaf extract of *M. Pigra* led to identification of five constituents. Among compounds separated, quercetin acts as an allelochemical compound by inhibiting the germination and emergence of *E. crus-galli.* It is major weed at paddy field, responsible for significant losses of rice yields [41]. Quercetin exhibited strongest inhibition to kill all weed seeds at the concentration of 1 mg/mL. With respect to the mode of action, quercetin like a flavonoid is related to interfere ATP production and to transport and degrade of auxin, resulting in dramatic alteration of the root morphogenic program of weeds [42]. This result proved that *M. Pigra* might be a suitable source for natural compounds and makes an important contribution to the application of these active ingredients in natural herbicide production in the future.

## 5 Conclusions

The findings of this investigation delineated that the leaf of *M. pigra* contains several plant growth inhibitors. The ethyl acetate solvent presented the highest inhibitory capability in comparison with hexane and water. The mixture of hexane and ethyl acetate in column chromatography successfully isolating and purifying lupeol, stigmastane-3,6-dione, quercetin, chrysoeriol, and daucosterol. Results in vitro bioassays indicated that quercetin possessed strongest in germination and growth of *Echinochloa crus-galli.* Further research is essential to detect and elucidate the mechanism of quercetin resisting against biochemical and physiological responses of some principal weeds, which could support the development of plant-based herbicides in agricultural production.

## Author contributions

Tran Ngoc Quy and Do Tan Khang: Conceived and designed the experiments; contributed reagents, materials, analysis tools or data and Wrote the paper.

Nguyen Phuc Dam and Nguyen Trong Tuan: Performed the experiments and wrote the paper.

Tran Thanh Men, Nguyen Van Ay and Nguyen Phuong Thuy: Analyzed and interpreted the data and Wrote the paper.

## Data availability statement

Data included in article/supp. material/referenced in article.

## Additional information

No additional information is available for this paper.

## Declaration of competing interest

The authors declare that they have no known competing financial interests or personal relationships that could have appeared to influence the work reported in this paper
